# Editorial: The impact of specific environmental exposures on breast, lung, and colon cancer: advancing public health strategies for enhanced outcomes

**DOI:** 10.3389/fpubh.2025.1483915

**Published:** 2025-02-14

**Authors:** Chitra Thakur, Uttara Saran, Fei Chen

**Affiliations:** ^1^Stony Brook Cancer Center and Department of Pathology, Renaissance School of Medicine, Stony Brook University, Stony Brook, NY, United States; ^2^Department of Genomic Medicine, The University of Texas MD Anderson Cancer Center, Houston, TX, United States

**Keywords:** environmental carcinogens, breast cancer, lung cancer, colon cancer, public health strategies, cancer risk

Cancer remains one of the most formidable public health challenges, with breast, lung, and colon cancer being the most prevalent and deadly cancer types worldwide (1, 2). It has become increasingly clear, that aside from genetic disposition, lifestyle choices and environmental factors have a profound impact on increasing an individual's risk of developing cancer (3–6). Exposure to harmful environmental agents—ranging from air pollutants to toxic chemicals—significantly influences cancer incidence, particularly in vulnerable populations (7). To effectively combat these cancers and reduce disparities among patients/survivors, public health strategies must be refined to address environmental risks, improve early detection, and ensure equitable access to care. This Research Topic focuses on advancing our understanding of the specific environmental exposures implicated in breast, lung, and colon cancer, primarily focusing on informing and advancing public health strategies. By exploring breakthrough information related to these cancers, we seek to uncover novel insights into the associations between these environmental exposures and their impact on carcinogenesis.

The relationship between environmental risk factors and cancer incidence is well-documented, yet it often lacks visibility in public discourse. Carcinogenic substances like tobacco, industrial pollutants, etc. are known to increase cancer incidence, particularly for lung, breast, and colon cancers. For example, prolonged exposure to air pollutants, such as particulate matter, has been directly linked to an increased incidence of lung cancer (8). Similarly, chemicals found in pesticides and plastics have been associated with breast cancer, while environmental influences on diet, such as the availability of processed foods, contribute to the incidence of colon cancer (9–11). In this Research Topic, several studies from various parts of the world as well as the United States present evidence of a growing risk of breast, colon, and lung cancer incidence due to prolonged exposure to environmental pollutants. Dos Santos et al. (12) in their study, showed that occupational exposure to pesticides in rural working women induced significant changes in the levels of cytokines necessary for tumor control and were positively correlated with worse prognostic outcomes. A meta-analysis study by Liu et al. (13) demonstrated significant associations between exposure to endocrine-disrupting chemicals (EDCs), which have the potential to interfere with the function of normal hormones, and an increased risk of breast cancer. They found that breast cancer risk was increased by exposure to certain EDC congeners and their metabolites, such as benzene, chlordane, hexachlorocyclohexane, and polychlorinated biphenyls. Similarly, Yuan et al. conducted a prospective cohort study to determine the relationship between Bisphenol A (BPA) exposure and cancer mortality. BPA, an environmental phenol, is utilized in various products, including baby bottles, and food containers (14), and has been shown to be detectable in more than 90% of urine samples in the general population in the United States (15), promoting some states to enforce regulations to restrict the use of BPA. The authors of this study determined that a lower level of BPA of <1.99 ng/mL was associated with a higher risk of cancer mortality. In their scoping review on military environmental exposures (MEE) including volatile organic compounds (VOCs), endocrine-disrupting chemicals (EDCs), tactile herbicides, airborne hazards and open burn pits (AHOBP), and depleted uranium on the risk of breast cancer among service members and Veterans, Jester et al. determined that MEE poses a unique risk to women veterans who were affected by MEE during their service. However, the authors concede that further studies are needed to validate these findings owing to the mixed and limited availability of literature on MEE and breast cancer among veterans.

Socio-economic demographics, resulting in higher carcinogen exposures and higher behavioral risk factors such as diet, physical activity, and obesity, or substance use such as smoking and alcohol consumption, also play integral roles in increasing cancer risk (16–19). For example, one-third of cancer deaths in the United States are attributed to diet, lack of physical activity, and obesity, while another third is correlated to exposure to tobacco products (20). In their perspective article, Atchade et al. highlight changes in Westernized dietary patterns in the United States as a significant contributor impacting the colonic microbiome and contributing to the recent surge of early-onset CRC (EOCRC). To determine the correlation between caffeine consumption and the prevalence of colon cancer, Qu et al. applied weighted logistic regression to the National Health and Nutrition Examination Survey (NHANES) dataset to evaluate correlations. They determined a potential dose–response relationship between an increased risk of colon cancer and higher caffeine intake levels. In continuation of their previous work demonstrating alcohol exposure selectively activates mammalian p38 mitogen-activated protein kinase (MAPK) in breast cancer cells, in their current study, Li et al. aimed to determine if Pirfenidone (PFD), an antifibrotic compound and pharmacological inhibitor of p38γ MAPK, could inhibit alcohol-induced promotion of breast cancer. Their results demonstrate that PFD successfully inhibited mammary tumor growth and alcohol-promoted metastasis, suggesting that this agent, which is currently approved for the treatment of idiopathic pulmonary fibrosis, could be re-purposed and used to treat aggressive breast cancer and alcohol-promoted mammary tumor progression.

It is also important to note that exposure to environmental carcinogens is not evenly distributed across populations, creating environmental inequity. Studies have shown that higher exposures to hazardous air pollutants as well as non-air-pollutant-related hazards, including water contaminants such as lead (21), lack of greenspace (22, 23), and poor walkability scores (24, 25) among socially and/or economically disadvantaged populations (26–32). An assessment of differences in colorectal cancer (CRC) survival between urban and rural areas by Fu et al., revealed a notable difference in CRC survival, highlighting the importance of considering urban–rural disparities in CRC prognosis and the influence of socioeconomic factors on survival outcomes. Higher total and CRC-specific mortality rates were found in rural areas as compared to urban areas. Interestingly, household incomes below $75,000 and $55,000 were found to be independent prognostic factors for the overall survival of CRC in urban and rural areas, respectively. The study also identified several independent prognostic factors influencing the overall survival of CRC patients, such as age over 40 years, male gender, black ethnicity, tumor location in the right colon, advanced stages (stage III and stage IV), and tumor size over 5 cm. To understand the impact of industrial installations such as steel plants, oil refineries urban discharges, etc.) two articles in the current Research Topic present their findings regarding correlations between residence in areas with high environmental pressures and death rates with a focus on female breast cancer characteristics (Giannico et al.) and bronchus/lung cancer characteristics (Mincuzzi et al.) respectively. Both studies found several independent prognostic factors for breast and lung cancer characteristics, respectively. While neither study was able to determine a clear association between these prognostic factors and living in the contaminated site of national interest (SIN) of Taranto, Italy, they did find a correlation between residential sites and an increased all-cause death rate. Interestingly, Mincuzzi et al. also found an association between male gender and a higher prevalence of poorly differentiated cancer and squamous-cell carcinoma. Finally, Zhao et al. sort to determine associations among incidence and mortality of Tracheal, Bronchus, and Lung (TBL) cancer, air pollutants, and greenspaces (which are known to improve air quality). The authors found positive associations between green spaces and air pollutants with TBL cancer, particularly among individuals aged 20 to 54. In summary, this study suggests that more green spaces/forests serve as protective factors, along with higher health care coverage, better health status, and participation in physical activities.

Despite the clear connection between environmental exposures and cancer incidence, public health efforts to mitigate these risks are often insufficient. This is especially concerning given that cancer survivors in underserved communities frequently face disparities in outcomes due to continued exposure to environmental hazards. Addressing these disparities requires a comprehensive approach that targets environmental risk factors and prioritizes the needs of vulnerable populations. Nolazco et al., in their cross-sectional study utilizing self-reported cancer histories from 39,578 participants in the Behavioral Risk Factor Surveillance System (BRFSS) database, found current and former smokers exhibited significantly poorer health-related quality of life (HRQoL) when compared to never smokers. These findings highlight the need to prioritize smoking cessation among cancer survivors. In conjunction, Tesfaw et al., in their systematic review to assess the comprehensive and common mortality-related risk factors of lung cancer, identified positive correlations between age, gender, stage, and comorbidities such as cardiovascular disease, hypertension, and diabetes on lung cancer mortality. In their nested case-control study, Xu et al. determined that prior history of chronic bronchitis, long-term wheezing symptoms, as well as exposure to environmental pollutants such as smoking, and biofuel combustion increased the risk of chronic obstructive pulmonary disease (COPD). Finally, Xiao et al.'s study investigating the epidemiological characteristics of lung cancer among healthcare workers in the Hunan Province, as well as the occupational risk factors, revealed that the prevalence of lung cancer among this cohort was much higher than that of the general population. Moreover, the prevalence of lung cancer was found to increase exponentially with age. In summary, this article highlights the occupational risks faced by general practitioners and medical imaging technicians, and the need to implement better personal safety measures.

Thus, addressing the impact of environmental exposure on breast, lung, and colon cancer requires a concerted effort from governments, public health officials, healthcare providers, and communities. By strengthening regulations, promoting environmental justice, enhancing public education, investing in research, and integrating environmental health into healthcare, we can advance public health strategies that lead to better outcomes for all. The fight against cancer is ongoing, but with a focus on environmental factors, we can make significant strides toward reducing its burden and improving the health and wellbeing of future generations.

In conclusion, the time is now for a proactive and comprehensive approach to addressing the environmental causes of cancer. By prioritizing this Research Topic within the broader public health agenda, we can move closer to a future where the incidence of breast, lung, and colon cancer is significantly reduced, and where all individuals have the opportunity to live in healthier environments.
